# A novel scoring system for stroke risk stratification in Japanese patients with low CHADS2 scores: Study using a transesophageal‐echocardiogram endpoint

**DOI:** 10.1002/joa3.12335

**Published:** 2020-04-06

**Authors:** Daigo Nagahara, Naoyuki Kamiyama, Takefumi Fujito, Atsushi Mochizuki, Shinya Shimoshige, Tetsuji Miura

**Affiliations:** ^1^ Department of Cardiovascular, Renal and Metabolic Medicine Sapporo Medical University School of Medicine Sapporo Japan; ^2^ Department of Cardiology Kushiro Kojinkai Memorial Hospital Kushiro Japan

**Keywords:** atrial fibrillation, CHADS2 score, spontaneous echo contrast, transesophageal echocardiography

## Abstract

**Background:**

Catheter ablation is an effective treatment for atrial fibrillation (AF), but it carries risk of perioperative thromboembolism even in cases with low CHADS2 scores. Here, we examined whether a combination of clinical variables can predict stroke risk factors that are assessed by transesophageal echocardiography (TEE).

**Methods:**

The study population consisted of 209 consecutive AF patients with a CHADS2 score of 0 or 1 (58.7 ± 10.6 years old; persistent AF, 33.0%). All patients underwent TEE, and TEE‐determined stroke risk (TEE risk) was defined as cardiac thrombus/sludge, dense spontaneous echo contrast (SEC), and/or peak left atrial appendage (LAA) flow velocity <0.25 m/s.

**Results:**

Transesophageal echocardiography risk was observed in 10.5% of the patients. In multivariate logistic analysis, persistent AF [odds ratio (OR): 11.5, CI: 3.14‐42.1, *P* = .0002], left atrial diameter (LAD) (OR: 1.10, CI: 1.01‐1.20, *P* = .0293), contrast medium defect (CMD) in the LAA detected by computed tomography (OR: 20.2, CI: 6.3‐65.0, *P* < .0001), and serum brain natriuretic peptide (BNP) level (OR: 1.00, CI: 1.00‐1.01, *P* = .0056) were independent predictors of TEE risk. A new scoring system comprising LAD > 41 mm (1 point), BNP > 47 pg/mL (1 point), CMD (2 points), and persistent AF (2 points) was constructed and defined as TEE‐risk score. The area under the curve (AUC) for prediction of TEE risk was 0.631 in modified CHADS2 score and it was 0.852 in TEE‐risk score.

**Conclusion:**

Transesophageal echocardiography risk is predictable by TEE‐risk score, and its combination with CHADS2 score may improve the stroke risk stratification in AF patients with a low CHADS2 score.

## Graphical abstract

Prevalence of transesophageal echocardiography (TEE)‐determined stroke risk according to each scoring system. TEE‐risk score had better discrimination power as measured by AUC compared to modified CHADS2 score.

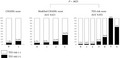

## 1 INTRODUCTION

Catheter ablation is an effective treatment for patients with atrial fibrillation (AF) and the number of procedures using catheter ablation has been increasing. One of the most important periprocedural complications is thromboembolism. Accurate risk stratification for stroke and appropriate anticoagulation management are therefore essential. The CHADS2 score is useful for stratifying the risk of stroke and thromboembolism in patients with AF and is widely used for assessment of the eligibility for anticoagulation therapy. It has a great advantage of not requiring an expensive medical examination, but its level of accuracy is reportedly modest. In a Japanese registry^1^ in which AF patients without anticoagulation therapy were enrolled, the annual incidences of stroke were 0.54% in patients with a CHADS2 score of 0% and 0.93% in patients with a CHADS2 score of 1. In another Japanese registry,^2^ patients with a low CHADS2 score (0 or 1) accounted for half of the total population of AF patients, suggesting that a low CHADS2 score group forms a considerable stroke source in the entire Japanese society despite the low annual incidence of stroke in Japan. Thus, for better stratification of stroke risk in AF patients, an index that improves risk assessment in patients with low CHADS2 scores is needed.

Several indices to complement the CHADS2 score for further risk stratification have been proposed. The indices include severity of fibrosis of the left atrium (LA) measured by magnetic resonance imaging,^3^ renal insufficiency,^4^ sleep apnea syndrome,^5^ biochemical markers such as high‐sensitivity troponin^6^, ^7^ and brain natriuretic peptide (BNP), specific morphology of the left atrial appendage (LAA),^8^ and P wave abnormality on a 12‐lead electrocardiogram.^9^ Transesophageal echocardiography (TEE) is useful for detecting left atrial thrombus, dense spontaneous echo contrast (SEC), and reduced peak LAA flow velocity, which are known to be associated with a high risk of thromboembolic events in patients with AF.^10^, ^11^, ^12^, ^13^, ^14^ However, TEE should not be recommended to all AF patients because of its invasive nature and possible complications.

Several surrogate markers based on left atrial dilatation or cardiac function have been proposed for risk stratification of cardiogenic stroke.^15^, ^16^, ^17^, ^18^, ^19^, ^20^ However, there has been no study in which those surrogate markers were analyzed in Japanese patients in whom catheter ablation was planned. The aim of this study was to determine simple and practical variables in addition to CHADS2 score that enable prediction of patients at high risk for stroke.

## 2 METHODS

The protocol for this research project was approved by the Ethics Committee of Sapporo Medical University (number 312‐73) and it conforms to the provisions of the Declaration of Helsinki.

### 2.1 Study population and clinical examinations

The study population consisted of 209 consecutive AF patients with a CHADS2 score of 0 or 1 who were scheduled to undergo catheter ablation during the period from 2009 to 2018 at our institute. Persistent AF was defined as continuous AF that was sustained for more than 7 days. Serum BNP and blood coagulation markers including fibrinogen (FBG), fibrinogen degradation products, d‐dimer, and thrombin‐antithrombin complex were measured at the time of admission. Enhanced computed tomography (CT) was performed 1 day before the catheter ablation, and three‐dimensional geography of the LA was created after resecting the distal pulmonary vein and LAA to calculate the LA volume. A contrast medium defect (CMD) in the LAA was diagnosed if the LAA was not filled with contrast medium and was not fully visualized by cardiac CT. Left atrial dimension (LAD), left ventricular ejection fraction (LVEF), and left ventricular end‐diastolic dimension were measured by transthoracic echocardiography. TEE was performed in a fasting condition on the morning of catheter ablation with a GE vivid 7 or ALOCA echocardiograph and a multiplane transesophageal probe (2.9‐7.0 MHz). The presence of cardiac thrombus or sludge, severity of SEC, and peak flow velocity of the LAA were determined. The gain was adjusted for optimal evaluation of SEC in the LA and LAA. SEC in the LA and LAA was defined as a pattern of slowly swirling smoke‐like echoes and was classified into four categories (absent, mild, moderate, and dense). Mild SEC is minimal SEC, transiently appearing during the cardiac cycle in the LAA or main cavity, and dense SEC is an intense swirling pattern of SEC in the LAA and main cavity that is constantly observed throughout the cardiac cycle. LAA peak flow velocity was measured by pulse‐wave Doppler echocardiographic imaging at the orifice of the LAA. TEE‐determined stroke risk (TEE risk) was defined as the presence of cardiac thrombus/sludge, dense SEC, or LAA peak flow velocity <0.25 m/s. Of the 209 patients, 202 patients were on anticoagulation therapy using warfarin or a direct oral anticoagulant (DOAC). Blood sampling and TEE were performed without interruption of oral anticoagulation therapy.

### 2.2 Indices of stroke risk

CHADS2 score was calculated for each patient at the time of TEE as follows: congestive heart failure (1 point) (defined as the presence of typical symptoms and/or treatment with diuretics, BNP > 100 pg/mL, and LVEF < 40%), hypertension (1 point), age ≧ 75 years (1 point), diabetes mellitus (1 point), and a history of stroke (2 points). In this study, we calculated the modified CHADS2 score by the addition of 1 point for one of the following risk factors proposed in Japanese guidelines for pharmacotherapy of AF^21^: vascular disease (prior myocardial infarction, aortic plaque, and peripheral arterial disease), cardiomyopathy, and age ≧ 65 years. We also calculated TEE‐risk score that consists of points for LAD > 41 mm (1 point), BNP > 47 pg/mL (1 point), presence of persistent AF (2 points), and CMD by cardiac CT (2 points) based on the results of logistic analysis for predictors of TEE risk (see Section 3).

### 2.3 Statistical analysis

Statistical values are shown as means ± 1SD. Patients were divided into two groups according to the presence or absence of TEE risk. The Mann‐Whitney *U* test was used for comparison of mean values in the two groups, and the chi‐square test was used for comparison of prevalence. A *P* value less than .05 was considered statistically significant. Binocular logistic analysis was used to identify independent predictors of TEE risk. Variables with a *P* value less than .05 in univariate analysis were entered into multivariate models. Receiver operating characteristic (ROC) curves were traced for the prediction of TEE risk using LAD and serum BNP level, and cutoff values were determined. Comparison of the diagnostic accuracies of modified CHADS2 score and TEE‐risk score for the prediction of TEE risk was performed using area under the curve (AUC) by ROC curve analysis. The analyses were performed using JMP software (version 8.0.2; SAS Institute).

## 3 RESULTS

### 3.1 Clinical characteristics of study subjects

The baseline characteristics of the study patients and comparison of clinical variables in the two groups with and without TEE risk are shown in Table 1. We enrolled 209 consecutive AF patients with a CHADS2 score of 0 (n = 97) or 1 (n = 112). The mean age of the patients was 58.7 ± 10.6 years, and 73.2% of the patients were male. Mean LAD was 38.9 ± 6.1 mm and only 3.3% of the patients had LAD > 50 mm. Mean LVEF was 60.5 ± 10.8% and only 4.3% of the patients had LVEF < 40%. The prevalence of persistent AF was 33.0% and mean duration of AF was 14.5 ± 14.1 months. Compared to patients with paroxysmal AF, those with persistent AF had lower LVEF (55.3 ± 12.0% vs 63.1 ± 9.1%, *P* < .0001), greater LAD (41.2 ± 6.3 mm vs 37.7 ± 5.6 mm, *P* = .0001), and higher BNP level (118.0 ± 151.4 pg/mL vs 64.9 ± 118.9 pg/mL, *P* = .0092). TEE risk was observed in 10.5% of the patients (no case with cardiac thrombus or sludge, dense SEC in 3.3% of the patients, and reduced LAA peak flow velocity in 9.6% of the patients). When clinical variables were compared in the group with TEE risk and the group without TEE risk, body mass index was higher (26.3 ± 4.0 vs 24.2 ± 3.2, *P* = .0047), modified CHADS2 score was higher (1.55 ± 1.01 vs 1.08 ± 0.87, *P* = .0351), heart failure was more frequent (36.4% vs 17.1%, *P* = .0428), prevalence of persistent AF was higher (81.8% vs 27.3%, *P* < .0001), LAD (43.4 ± 6.3 mm vs 38.3 ± 5.8 mm, *P* = .0008), and CT volume of the LA (126 ± 26 mL vs 100 ± 31 mL, *P* = .0002) were greater, prevalence of CMD was higher (50.0% vs 4.8%, *P* < .0001), and serum levels of BNP (182 ± 228 pg/mL vs 69 ± 109 pg/mL, *P* = .0009) and fibrinogen (309 ± 54 mg/dL vs 281 ± 60 mg/dL, *P* = .0126) were higher in patients with TEE abnormality. There was no significant difference in LVEF, prevalence of cardiomyopathy, and prescription of anticoagulation therapy between the two groups. In patients on warfarin, averaged prothrombin time international normalized ratio at the time of TEE was 2.20 ± 0.50, and 69.2% of the patients were in the therapeutic range. A low‐dose DOAC was prescribed in 19.0% (31/163) of the patients and the low dose was an inappropriate dose in 8.6% (14/163) of the patients.

**TABLE 1:** Baseline clinical characteristics (n = 209) and comaprison between patients with TEE risk and without

	Total (n = 209)	TEE risk (+) (n = 22)	TEE risk (−) (n = 187)	*P*‐value
Age (y)	58.7 ± 10.6	62.0 ± 10.3	58.3 ± 10.6	.0781
Male	153 (73.2%)	16 (72.7%)	137 (76.1%)	1
Height (cm)	166.5 ± 9.9	166.3 ± 11.7	166.6 ± 9.7	.9100
Weight (kg)	68.2 ± 13.4	73.3 ± 16.2	67.6 ± 12.9	.0597
Body mass index	24.4 ± 3.4	26.3 ± 4.0	24.2 ± 3.2	.0047*
CHDAS2 score 0/1	97/112	7/15	90/97	.1780
CHDAS2 score	0.54 ± 0.50	0.69 ± 0.48	0.52 ± 0.50	.1480
Modified CHADS2 score	1.13 ± 0.89	1.55 ± 1.01	1.08 ± 0.87	.0351*
Heart failure	40 (19.1%)	8 (36.4%)	32 (17.1%)	.0428*
Hypertension	63 (30.1%)	7 (31.8%)	59 (31.6%)	1
Age ≧ 75	0 (0%)	0 (0%)	0 (0%)	1
Diabetes mellitus	6 (2.9%)	0 (0%)	6 (3.2%)	1
MI or PAD	13 (6.2%)	1 (4.8%)	12 (6.4%)	1
Cardiomyopathy	31 (14.8%)	6 (27.3%)	25 (13.4%)	.1080
Age 65‐74	79 (37.8%)	12 (54.5%)	67 (35.8%)	.1050
Persistent AF	69 (33.0%)	18 (81.8%)	51 (27.3%)	.0001*
LAD (mm)	38.9 ± 6.1	43.4 ± 6.3	38.3 ± 5.8	.0008*
EF (%)	60.5 ± 10.8	57.9 ± 11.6	60.8 ± 10.7	.2220
LVDd (mm)	46.8 ± 6.1	47.9 ± 6.8	46.6 ± 6.0	.4840
TMF E (m/s)	0.65 ± 0.17	0.76 ± 0.22	0.64 ± 0.16	.0207*
TMF A (m/s)	0.58 ± 0.19	0.55 ± 0.30	0.59 ± 0.18	.5570
LAAPV (m/s)	0.59 ± 0.25	0.21 ± 0.05	0.63 ± 0.23	<.0001*
SEC	0.63 ± 0.82	1.78 ± 1.07	0.49 ± 0.67	<.0001*
LA volume by CT (mL)	103 ± 32	126 ± 26	100 ± 31	.0002*
Contrast medium defect	20 (9.6%)	11 (50.0%)	9 (4.8%)	<.0001*
BNP (pg/mL)	81 ± 131	182 ± 228	69 ± 109	.0009*
Cr (mg/dL)	0.84 ± 0.17	0.87 ± 0.12	0.83 ± 0.18	.2440
FBG (mg/dL)	284 ± 60	309 ± 54	281 ± 60	.0126*
FDP (μg/mL)	3.01 ± 1.02	3.21 ± 1.12	2.99 ± 1.00	.3530
D‐dimer (μg/mL)	0.41 ± 0.50	0.54 ± 0.73	0.40 ± 0.47	.6600
TAT (ng/mL)	1.92 ± 2.95	1.74 ± 1.48	1.94 ± 3.08	.9760
PT‐INR	2.20 ± 0.50	2.11 ± 0.35	2.22 ± 0.53	.6340
Warfarin in therapeutic range	27/39 (69.2%)	5/6 (83.3%)	22/33 (66.7%)	.6450
Anticogulants				.3350
Dabigatran	38/209 (18.2%)	5/22 (22.7%)	33/187 (17.6%)	
Rivaroxaban	53/209 (25.4%)	5/22 (22.7%)	48/187 (25.7%)	
Apixaban	48/209 (23.0%)	5/22 (22.7%)	43/187 (23.0%)	
Edoxaban	24/209 (11.5%)	0/22 (0%)	24/187 (12.9%)	
Low‐dose DOAC	31/163 (19.0%)	1/15 (6.7%)	30/148 (20.3%)	.3070
Inappropriate low dose DOAC	14/163 (8.6%)	0/15 (0%)	14/148 (9.5%)	.3670
Warfarin	39/209 (18.7%)	6/22 (27.3%)	33/187 (17.6%)	

Abbreviations: AF, atrial fibrillation; BNP, brain natriuretic peptide; CT, computed tomography; DOAC, direct oral anticoagulant; EF, ejection fraction; FBG, fibrinogen; FDP, fibrinogen degradation products; LA, left atrium; LAAPV, left atrial appendage peak velocity; LAD, left atrial diameter; LVDd, left ventricular end‐diastolic diameter; MI, myocardial infarction; PAD, periferal arterial disease; PT‐INR, prothrombin time international normalized ratio; SEC, spontaneous echo contrast; TAT, thrombin‐antithrombin complex; TEE, transesophageal echocardiogram; TMF A, transmitral flow velocity during late phase of diastole; TMF E, transmitral flow velocity during early phase of diastole.

* *P* < .05.

### 3.2 Clinical parameters associated with TEE risk

**FIGURE 1:**
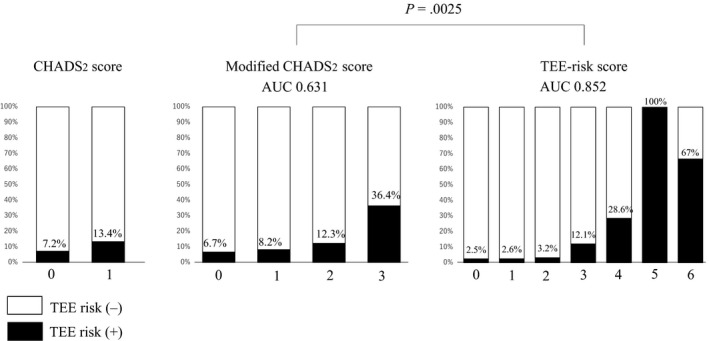
Prevalence of transesophageal echocardiography (TEE)‐determined stroke risk according to each scoring system

The results of univariate and multivariate analyses using a binocular logistic regression model for prediction of TEE risk are shown in Table 2. In univariate analysis, body mass index (odds ratio [OR]: 1.20, confidence interval [CI]: 1.05‐1.37, *P* = .0061), modified CHADS2 score (OR: 1.81, CI: 1.08‐3.01, *P* = .0235), heart failure (OR: 2.77, CI: 1.07‐7.15, *P* = .0354), persistent AF (OR: 12.0, CI: 3.88‐37.2, *P* < .0001), LAD (OR: 1.15, CI: 1.06‐1.24, *P* = .0009), peak transmitral flow velocity during the early phase of diastole (OR: 1.03, CI: 1.01‐1.06, *P* = .0053), CT volume of the LA (OR: 1.02, CI: 1.01‐1.04, *P* = .0021), CMD (OR: 21.6, CI: 7.13‐65.4, *P* < .0001), serum BNP level (OR: 1.00 per 1 pg/mL, CI: 1.00‐1.01, *P* = .0046), and fibrinogen level (OR: 1.01, CI: 1.00‐1.01, *P* = .0381) were associated with TEE risk. In multivariate analysis, persistent AF (OR: 11.5, CI: 3.14‐42.1, *P* = .0002), LAD (OR: 1.10, CI: 1.01‐1.20, *P* = .0293), CMD (OR: 20.2, CI: 6.3‐65.0, *P* < .0001), and serum BNP level (OR: 1.00, CI: 1.00‐1.01, *P* = .0056) were independently associated with TEE risk. Optimal cutoff values for the prediction of TEE risk were 41 mm for LAD and 47.4 pg/mL for serum BNP level. The AUC values for prediction of TEE risk were 0.631 in modified CHADS2 score and 0.852 in TEE‐risk score. TEE‐risk score had higher discrimination power as measured by AUC than that of modified CHADS2 score (*P* = .0025) (Figure 1). The optimal cutoff value of TEE‐risk score for prediction of TEE risk was 3 points, and its sensitivity and specificity were 81.8% and 77.6%, respectively. Subgroup analysis was performed in 20 patients with CMD; the mean TEE‐risk score tended to be higher in patients with TEE risk than in patients without TEE risk (5.5 ± 0.7 vs 4.2 ± 1.5, *P* = .059). TEE‐risk score predicted the presence of TEE risk in patients with CMD (AUC: 0.742, sensitivity: 66.7%, and specificity: 90.9%) and the optimal cutoff value was 5 points.

**TABLE 2:** Uni and multivariate analysis for prediction of TEE risk

	Univariate anlysis				Multivariate anlysis		
	OR		95% CI	*P* value	OR	95% CI	*P* value
Age	1.04		0.99‐1.09	.1210			
Male	0.97		0.36‐2.63	.9570			
Height (cm)	1.00		0.95‐1.04	.9100			
Weight (kg)	1.03		1.00‐1.06	.0626			
Body mass index	1.20		1.05‐1.37	.0061*			
CHDAS2 score	1.99		0.78‐5.10	.1530			
Modified CHADS2 score	1.81		1.08‐3.01	.0235*			
Heart failure	2.77		1.07‐7.15	.0354*			
Hypertension	1.01		0.39‐2.61	.9800			
Diabetes mellitus	0.000000526		0	.9880			
MI or PAD	0.69		0.09‐5.61	.7320			
Cardiomyopathy	2.43		0.87‐6.80	.0906			
Age ≧ 65	2.15		0.88‐5.24	.0923			
Persistent AF	12.0		3.88‐37.2	<.0001*	11.5	3.14‐42.1	.0002*
LAD (mm)	1.15	(per 1 mm)	1.06‐1.24	.0009*	1.10	1.01‐1.20	.0293*
EF (%)	0.98	(per 1.0%)	0.94‐1.02	.2530			
LVDd (mm)	1.03		0.96‐1.11	.3750			
TMF E (m/s)	1.03	(per 0.01 m)	1.01‐1.06	.0053*			
TMF A (m/s)	0.99	(per 0.01 m)	0.94‐1.03	.5990			
LA volume by CT (mL)	1.02	(per 1mL)	1.01‐1.04	.0021*			
Contrast medium defect	21.6		7.13‐65.4	<.0001*	20.2	6.3‐65.0	<.0001*
BNP (pg/mL)	1.00	(per 1 pg/mL)	1.00‐1.01	.0046*	1.00	1.00‐1.01	.0056*
Cr (mg/dL)	3.25		0.28‐37.9	.3470			
FBG (mg/dL)	1.01		1.00‐1.01	.0381*			
FDP (μg/mL)	1.21		0.82‐1.78	.3390			
D‐dimer (μg/mL)	1.44		0.78‐2.65	.2410			
TAT (ng/mL)	0.97		0.80‐1.18	.7700			
PT‐INR	0.63		0.10‐3.95	.6250			
Warfarin in therapeutic range	2.50		0.26‐24.1	.4280			
Anticogulants							
Dabigatran	1.37		0.47‐3.98	.0560			
Rivaroxaban	0.85		0.30‐2.43	.7640			
Apixaban	0.99		0.34‐2.82	.9770			
Edoxaban	<0.01		—	.9900			
Low‐dose DOAC	0.28		0.04‐2.22	.2290			
Inappropriate low dose DOAC	<0.01		—	.9930			
Warfarin	1.75		0.64‐4.81	.2780			

Abbreviations: CI, confidence interval; OR, odds ratio; other abbreviations are the same as those in Table 1.

* *P* < .05.

## 4 DISCUSSION

### 4.1 Prevalence of patients at high risk for stroke despite having a low CHADS2 score

It has been reported that cardiac thrombus/sludge, dense SEC, and reduced LAA peak flow velocity in TEE can be observed in a low percentage of patients with a low CHADS2 score of 0 or 1.^12^, ^16^, ^17^, ^18^ Kleemann et al^16^ enrolled 295 patients with a CHADS2 score of 0 or 1 who underwent TEE before electrical cardioversion (structural heart disease in 45%, LVEF < 40% in 12.9%, and LAD > 50 mm in 10.2%), and cardiac thrombus and dense SEC were detected in 3% and 8% of the patients, respectively. Puwanant et al^18^ reported a similar rate of cardiac thrombus in patients with a CHADS2 score of 0 or 1 who underwent TEE before AF ablation (0% in patients with a CHADS2 score of 0 and 3% in patients with a CHADS2 score of 1). In a study by Sasahara et al^12^ that included 280 AF patients with a CHADS2 score of 0 or 1 (mean age of 64 years and 48% of the patients having persistent AF), LA abnormality defined as dense SEC or reduced LAA peak flow velocity was observed in 24% of the patients. Providencia et al^17^ defined LA abnormality as cardiac thrombus, dense SEC, and/or reduced LAA peak flow velocity (<0.20 m/s). The incidences of LA abnormality were 8.3% in patients with a CHADS2 score of 0 and 21.5% in patients with a CHADS2 score of 1. In the present study, there was no patient with cardiac thrombus or sludge, but TEE risk (defined as dense SEC and/or LAA peak flow velocity <0.25 m/s) was observed in 10.5% of the patients. The rates of abnormal findings in the LA were lower in this study than in previous studies, and the lower rates would be attributable to clinical characteristics of the study population (younger, smaller LA, and lower percentage of patients with persistent AF than those in earlier studies). However, it is notable that even in the present study population with mild LA remodeling, a relatively large percentage of patients (10.5%) showed TEE signs of high cardiogenic stroke risk.

### 4.2 Clinical significance of risk stratification for stroke prior to catheter ablation

Catheter ablation is a safe and effective treatment for patients with AF, but it carries the risk of periprocedural thromboembolism. Periprocedural major stroke is rare, but silent stroke does not appear to be rare. In recent studies, silent stroke was detected by brain magnetic resonance imaging after catheter ablation in 9.6%~27.2% of patients,^22^, ^23^ and Nagao et al reported transient elevation of coagulation markers after catheter ablation.^24^ Optimal anticoagulation management to minimize periprocedural complications in patients undergoing catheter ablation has not been established.^24^, ^25^, ^26^ Hence, we attempted to improve thrombotic risk stratification in patients scheduled for AF ablation by data including TEE and CT. Detailed stratification of AF risk is important before catheter ablation, but it may also be useful for determining the timing of discontinuation of anticoagulation therapy after catheter ablation.

### 4.3 Surrogate markers for stroke risk detectable by TEE

Although TEE is useful for risk stratification in patients with AF,^10^, ^11^, ^12^, ^13^, ^14^ it should not be recommended to all patients because of its invasive nature. Several indices that can be derived from TTE have been proposed as surrogate markers for cardiac thrombus and dense SEC in TEE^16^, ^17^, ^18^: EF < 40% or LAD > 50 mm,^16^ reduced LVEF and LA dilatation measured as the LA area,^17^ and past history of heart failure and LVEF < 35%.^18^ The clinical benefit of serum N‐terminal pro‐BNP for embolic risk was also reported^6^ in patients on anticoagulation therapy. In the analysis, serum N‐terminal pro‐BNP level successfully stratified the risk of stroke or systemic embolism in addition to the CHA2DS2‐VASc score, and the minimum cutoff value was 363 ng/L. In the present study, cutoff values for TEE‐risk prediction were 47 pg/mL for BNP and 41 mm for LAD, which were lower than previous results possibly due to the younger study population and mild LA remodeling. Multidetector CT is commonly used before catheter ablation of AF and has been reported to be sensitive for detection of cardiac thrombus.^27^ These indices can be determined simply and at low cost and are thus feasible in daily clinical practice.

### 4.4 Difference in stroke risk according to type of AF

There has been controversy regarding the difference between stroke risk in patients with paroxysmal AF and that in patients with persistent AF. Although several studies demonstrated that stroke risk in patients with persistent AF was higher than that in patients with paroxysmal AF,^28^, ^29^ it was concluded from analysis of data in a Japanese registry^30^ that stroke risk of paroxysmal AF and that of persistent AF were similar after adjustment of the CHADS2 score. In the present study, persistent AF was a strong predictor of TEE risk. Patients with persistent AF in the present study had significantly greater LAD, higher BNP level, and lower LVEF than those in patients with paroxysmal AF, and the advanced atrial remodeling in patients with persistent AF might have augmented the significance of persistent AF.

### 4.5 Validity of TEE‐risk score in patients with and those without anticoagulation therapy

In the present study, 96.7% of the patients were on anticoagulation therapy at the time of TEE to prevent a periprocedural thromboembolic event. Anticoagulation therapy can modify levels of serum anticoagulation markers such as d‐dimer and FDP, and it potentially reduces the predictive value of these indices. However, earlier studies^31^ have demonstrated that anticoagulation therapy did not affect the severity of SEC or LAA flow velocity. Anticoagulation therapy does not directly affect ventricular function or its loading conditions and thus BNP level. Since anticoagulation therapy does not directly modulate any of the components of the score system and reduces embolic events, the predictive value of TEE‐risk score is unlikely be reduced in patients with anticoagulation therapy.

Since the validity of TEE‐risk score as a predictor of a thromboembolic event has not been confirmed by a prospective study using the event as a primary endpoint, it is obviously too early to use TEE‐risk score for indication of anticoagulation therapy. However, it is reasonable to use TEE‐risk score for guiding further examinations (such as brain MRI for screening silent stroke and TEE) in patients with a low CHADS2 score. TEE‐risk score >3 predicts TEE risk with sensitivity of 81.8% and specificity of 77.6%, which may justify TEE even when CHADS2 score is <2.

### 4.6 Limitations

First, we used TEE risk as an endpoint in this study since SEC and reduced LAA peak flow velocity detected by TEE have been shown to be associated with future stroke in previous studies.^10^, ^11^, ^12^, ^13^, ^14^ However, there was no case with cardiac thrombus or sludge in the present study. We need a long‐term follow‐up to detect future cardiac thrombus or sludge. Second, although we used SEC as one of the risk factors of stroke, SEC is known to be exacerbated just after the restoration of sinus rhythm and to improve with time in patients with PAF. Even if SEC was absent or mild at the time of TEE, severe SEC might have developed just after the termination of AF, and we might therefore have underestimated the stroke risk in patients with PAF. Third, LAA peak flow velocity is known to be different during sinus rhythm and AF, and the optimal cutoff value for prediction of stroke risk might be different depending on the cardiac rhythm, though we could not perform such subgroup analysis because of the small number of study subjects.

## 5 CONCLUSION

Even in patents with a low CHADS2 score of 0 or 1, approximately 10% of the patients have TEE signs of high cardiogenic stroke risk, which may be predicted by dilatation of the LA in TTE, serum BNP level, enhanced cardiac CT, and type of AF.

## CONFLICT OF INTEREST

The authors declare no conflict of interests for this article.
